# Elucidating the Role of Ezh2 in Tolerogenic Function of NOD Bone Marrow-Derived Dendritic Cells Expressing Constitutively Active Stat5b

**DOI:** 10.3390/ijms21186453

**Published:** 2020-09-04

**Authors:** Echarki Zerif, Farhan Ullah Khan, Ahmed Aziz Raki, Véronique Lullier, Denis Gris, Gilles Dupuis, Abdelaziz Amrani

**Affiliations:** Department of Pediatric, Immunology division, Centre de Recherche Clinique du CHUS, Faculty of Medicine and Health Sciences, University of Sherbrooke, 3001-12th Avenue North, Sherbrooke, QC J1H 5N4, Canada; echarki.zerif@umontreal.ca (E.Z.); Farhan.Ullah.Khan@USherbrooke.ca (F.U.K.); Ahmed.Aziz.Raki@USherbrooke.ca (A.A.R.); Veronique.Lullier@USherbrooke.ca (V.L.); denis.gris@usherbrooke.ca (D.G.); gilles.dupuis@usherbrooke.ca (G.D.)

**Keywords:** tolerogenic dendritic cells, Interferon Regulatory Factor 4 and 8, Stat5b transcription factor, histone methyltransferase, nonobese diabetic mice, type 1 diabetes

## Abstract

Tolerogenic dendritic cells (toDCs) are crucial to controlling the development of autoreactive T cell responses and the prevention of autoimmunity. We have reported that NOD.CD11c^Stat5b-CA^ transgenic mice expressing a constitutively active (CA) form of *Stat5b* under the control of a CD11c promoter are protected from diabetes and that Stat5b-CA-expressing DCs are tolerogenic and halt ongoing diabetes in NOD mice. However, the molecular mechanisms by which Stat5b-CA modulates DC tolerogenic function are not fully understood. Here, we used bone marrow-derived DCs (BMDCs) from NOD.CD11c^Stat5b-CA^ transgenic mice (Stat5b-CA.BMDCs) and found that Stat5b-CA.BMDCs displayed high levels of MHC class II, CD80, CD86, PD-L1, and PD-L2 and produced elevated amounts of TGFβ but low amounts of TNFα and IL-23. Stat5b-CA.BMDCs upregulated *Irf4* and downregulated *Irf8* genes and protein expression and promoted CD11c^+^CD11b^+^ DC2 subset differentiation. Interestingly, we found that the histone methyltransferase Ezh2 and Stat5b-CA bound gamma-interferon activated site (GAS) sequences in the *Irf8* enhancer IRF8 transcription, whereas Stat5b but not Ezh2 bound GAS sequences in the *Irf4* promoter to enhance IRF4 transcription. Injection of Stat5b-CA.BMDCs into prediabetic NOD mice halted progression of islet inflammation and protected against diabetes. Importantly, inhibition of Ezh2 in tolerogenic Stat5b-CA.BMDCs reduced their ability to prevent diabetes development in NOD recipient mice. Taken together, our data suggest that the active form of Stat5b induces tolerogenic DC function by modulating IRF4 and IRF8 expression through recruitment of Ezh2 and highlight the fundamental role of Ezh2 in Stat5b-mediated induction of tolerogenic DC function.

## 1. Introduction

Dendritic cells (DCs) are professional antigen-presenting cells (APCs) that are essential for the induction of effective immunity and the maintenance of immune tolerance. These opposite DC functions depend on their state of maturation, as well as their anatomical location [1]. Several lines of evidence suggest that immature and not fully mature DCs (semi-mature DCs) possess tolerogenic properties [2]. In contrast, it is generally thought that fully matured DCs expressing high levels of MHC class II, co-stimulatory markers (CD80 and CD86), and pro-inflammatory cytokines (IL-12p70, IL-23, and TNFα) are determinants required for the efficient induction of T effector cell responses. However, the outcome of DC maturation does not result all the time in the generation of DCs with immunogenic properties but may induce tolerogenic properties, depending on the nature of the immunogenic or tolerogenic signal as well as the involvement of transcriptional factors. Some danger-associated molecular patterns and immunosuppressive cytokines have been shown to drive the maturation of DCs that possess tolerogenic properties [3,4]. These fully mature tolerogenic DCs (toDCs), along with expression of co-stimulatory molecules, also express co-inhibitory molecules such as programmed death ligands PD-L1 and PD-L2 and immunoglobulin-like transcript 3 (ILT3). toDCs halt the expression of pro-inflammatory cytokines and produce immunosuppressive cytokines such as IL-10 and TGF-*β.* Therefore, DCs are able to exert their tolerogenic actions using different mechanisms, including induction of T-cell anergy, clonal deletion [5,6], and Treg differentiation. For example, Treg differentiation induced by toDCs has been shown to be mediated through membrane-bound PD-L1, which blocks the Akt/mTOR pathway to preferentially promote naive T cells to become Tregs [7]. Furthermore, secreted cytokines such as IL-10, IL-27, and TGF-*β*, as well as retinoic acid and IDO, have been shown to be able to convert naive CD4^+^ T cells into Tregs.

Transcriptional regulatory mechanisms involved in orchestrating the immunogenic and tolerogenic function of DCs are beginning to emerge but are far from being fully understood. We used the autoimmune mouse model NOD in which mature DCs are more prone to be immunogenic. In this model, the autoimmune response is due, at least in part, to molecular alterations in the Stat5b signaling pathway. It has been reported that DCs of NOD mice carry a *Stat5b* mutation at the first residue of the DNA-binding domain, which results in weak Stat5b DNA binding and reduced expression of downstream genes located upstream of the GAS consensus sequences [8]. To overcome this Stat5b defect, we generated a transgenic NOD model that carries an active form of Stat5b from the non-prone diabetic mouse C57BL/6. We have reported that overexpression of Stat5b-CA in DCs reprograms the cells to acquire tolerogenic functions that induce and maintain protective immune response against type 1 diabetes in NOD mice [9]. In the present study, we used an in vitro culture system of bone marrow-derived DCs (BMDCs) from NOD.CD11c^Stat5b-CA^ mice (Stat5b-CA.BMDCs) and NOD mice (BMDCs) to investigate the molecular mechanism that drives mature DCs’ tolerogenic and immunogenic functions. We found that Stat5b-CA.BMDCs expressed high levels of CD80, CD86, CD40, MHC class II as well as inhibitory molecules PD-L1 and PD-L2 as compared to immunogenic NOD BMDCs. Stat5b-CA.BMDCs produced high amounts of the anti-inflammatory cytokine TGFβ but low amounts of pro-inflammatory cytokines TNFα and IL-23. Stat5b-CA expression upregulated *Irf4* and downregulated *Irf8* gene expression while promoting CD11c^+^CD11b^+^ DC subset differentiation. Interesting, we found that the Ezh2 methyltransferase interacted with the Stat5b complex that bound GAS sequences in the *Irf8* enhancer. In contrast, Ezh2 did not interact with GAS sequences in the *Irf4* promoter. Of significance, a single injection of Stat5b-CA.BMDCs into 7- to 8-week-old NOD mice protected the animal from type 1 diabetes, whereas the transfer of Stat5b-CA.BMDCs in which Ezh2 was inhibited have a reduced ability to protect against diabetes. This study revealed for first time the fundamental role of the Ezh2 methyltransferase in Stat5b-induced DC tolerogenic function.

## 2. Results

### 2.1. Bone Marrow-Derived DCs from NOD.CD11c^Stat5b-CA^ Mice Display the Signature of Tolerogenic Mature DCs

**Figure 1:**
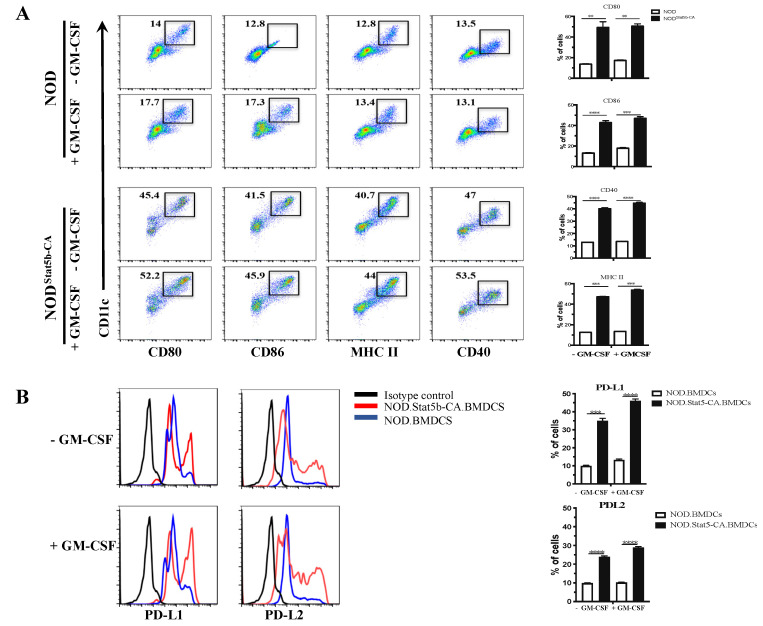
Stat5b-CA.BMDCs display tolerogenic properties. Bone marrow-derived dendritic cells (BMDCs) (1 × 10^5^ cells/well) were generated from NOD and NOD.CD11c^Stat5b-CA^ mice and cultured for 48 h in the absence or presence of granulocyte-macrophage colony-stimulating factor (GM-CSF) (50 ng/mL). (**A**) Cell surface expression of CD80, CD86, CD40, and MHC II. Numbers in the windows correspond to the percentages of positive cells with respect to total population of BMDCs. (**B**) Expression of PD-L1 and PD-L2 on CD11c^+^ BMDCs was determined by multicolor flow cytometry analysis in cells exposed or not to GM-CSF. Data are representative of three independent experiments. The asterisks indicate statistically significant differences determined by one-way ANOVA tests. *p* < 0.01 (**), *p* < 0.001 (***), and *p* < 0.0001 (****).

The capacity of DCs to promote immune tolerance or inflammatory immune responses is directly associated with their state of maturation. Several lines of evidence support the notion that mature DCs display the duality of APCs, being capable of linking innate and adaptive immunity or inducing immune tolerance to specific antigens. We have reported that LPS-activated splenic DCs of NOD.CD11c^Stat5b-CA^ mice displayed a mature phenotype and acquired tolerogenic DC signatures [9]. To further understand the molecular mechanisms underlying mature DC tolerogenic function, we used in vitro BMDCs generated by a combination of Granulocyte-Macrophage Colony-Stimulating Factor (GM-CSF) and IL-4, a condition that induces the generation of conventional DCs. To determine their maturation status, BMDCs were generated from 6- to 8-week-old NOD and NOD.CD11c^Stat5b-CA^ mice and cultured in the absence or presence of GM-CSF (50 ng/mL) for 48 h. Expression of co-stimulatory molecules was analyzed by flow cytometry (FACS). Results showed that unstimulated and GM-CSF-stimulated Stat5b-CA.BMDCs expressed high levels of MHC-II, CD80, CD86, and CD40 co-stimulatory molecules as compared to BMDCs of NOD mice (Figure 1A). Since tolerogenic DCs have been shown to promote central or peripheral tolerance through different mechanisms, including the expression of PD-L1 and PD-L2 [10,11], expression of these two immunomodulatory molecules was determined by FACS. Results showed that unstimulated CD11c^high^ Stat5b-CA.BMDCs expressed greater levels of PD-L1 and PD-L2 than BMDCs of NOD mice and were highly upregulated as a result of GM-CSF stimulation (Figure 1B). However, levels of PD-L1 and PD-L2 on CD11c^high^ BMDCs of NOD mice remained low, before and after GM-CSF stimulation (Figure 1B). These data were taken as evidence that CD11c^high^ Stat5b-CA.BMDCs expressed high levels of CD80, CD86, and MHC class II markers as compared to NOD BMDCs and that Stat5b-CA.BMDCs upregulated their expression of inhibitory molecules PD-L1 and PD-L2 following GM-CSF stimulation.

### 2.2. Pattern of Cytokine Gene Expression and Production Displayed by Stat5b-CA.BMDCs

**Figure 2:**
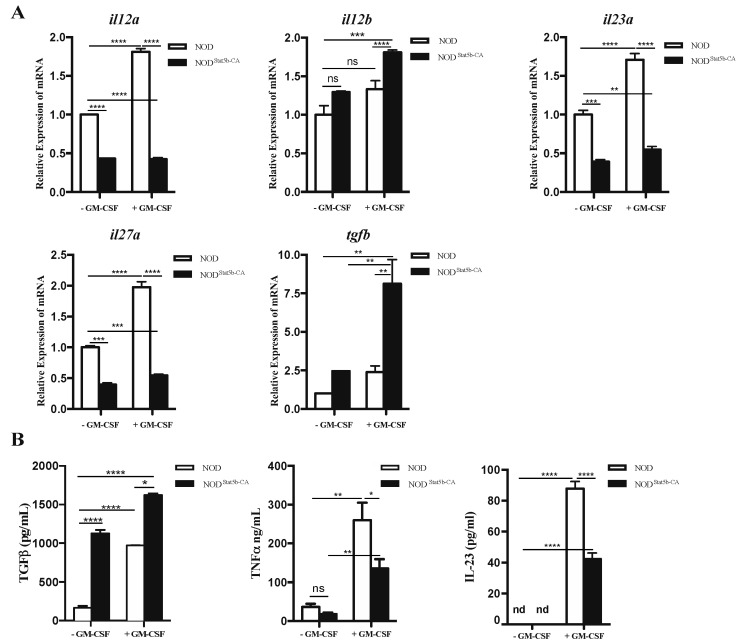
Cytokine profiles of BMDCs derived from NOD and NOD.CD11c^Stat5b-CA^ mice. BMDCs (1 × 10^5^ cells/well) generated from NOD and NOD.CD11c^Stat5b-CA^ mice were cultured for 48 h in the absence or presence of GM-CSF (50 ng/mL). (**A**) Relative expression of *Il12a*, *Il12b*, *Il23a*, *Il27a*, and *Tgfβ* genes was determined by qPCR using the ΔΔCT method. qPCR data are shown as relative expression compared to untreated BMDCs of NOD mice. (**B**) Quantification of TGFβ, TNFα, and IL-23 released in the supernatants of BMDCs exposed or not to GM-CSF as determined by ELISA. Data are shown as the mean ± SEM of at least three independent experiments. The asterisks indicate statistically significant differences determined using one-way ANOVA with Tukey’s post-test. *p* < 0.05 (*), *p* < 0.01 (**), *p* < 0.001 (***), and *p* < 0.0001 (****). nd: not detected.

The tolerogenic state of DCs is essentially characterized by their capacity to enhance expression of immunosuppressive cytokines while reducing their production of pro-inflammatory cytokines. To assess the pattern of cytokine expression in Stat5b-CA.BMDCs, we analyzed the expression of the pro-inflammatory cytokines TNFα and IL-23 and the anti-inflammatory cytokine TGFβ at the mRNA and protein levels. Results of qPCR showed that *Il12a*, *Il23a*, and *Il27a* but not *Il12b* gene expression was reduced, whereas *Tgfβ* gene expression was increased in unstimulated and stimulated Stat5b-CABMDCs compared to BMDCs of NOD mice (Figure 2A). Consistent with real-time PCR results, quantification of cytokines showed that Stat5b-CA.BMDCs produced higher amounts of TGFβ but lower amounts of TNFα and IL-23 than BMDCs of NOD mice (Figure 2B). These results provided clear evidence that Stat5b-CA-expressing BMDCs of NOD mice switched their pro-inflammatory cytokine profile to an anti-inflammatory cytokine set.

### 2.3. Tolerogenic Stat5b-CA.BMDCs Induce a Long-Term Immune Tolerance In Vivo

**Figure 3:**
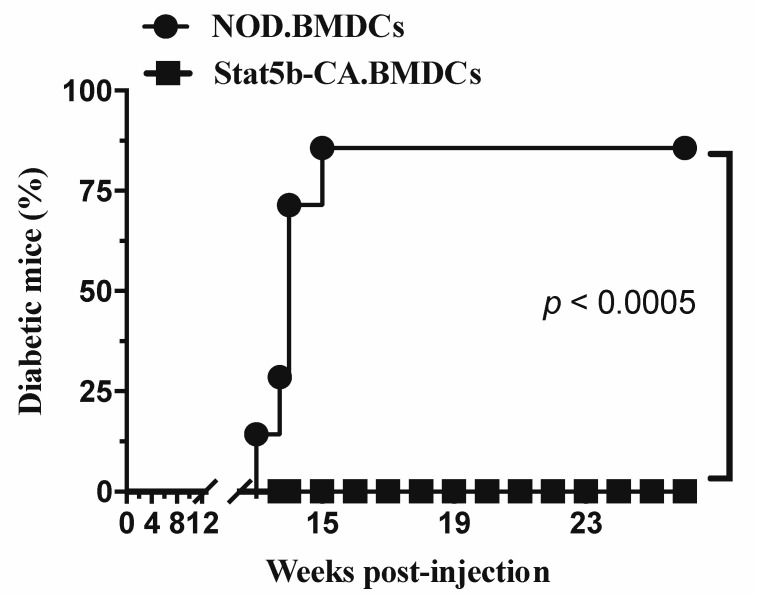
Inhibition of diabetes development by Stat5b-CA.BMDC treatment. Female 8- to 9-week-old NOD mice (7 mice per group) received one i.v. injection of BMDCs (10^7^ cells/mouse) derived from NOD or NOD.CD11c^Stat5b-CA^ mice. The animals were followed for diabetes development until 36 weeks (26 weeks post-injection) of age. For comparison of diabetes incidence between different groups, statistical analysis was performed with the Gehan–Breslow–Wilcoxon test.

To validate the capacity of tolerogenic Stat5b-CA.BMDCs to induce antigen-specific immune tolerance in vivo, we investigated their ability to induce immune tolerance in an autoimmune disease setting such as halting ongoing autoimmune diabetes in diabetes-prone NOD mice. Experimentally, prediabetic 8- to 9-week-old prediabetic NOD mice were injected intravenously with BMDCs generated from NOD or NOD.CD11c^Stat5b-CA^ mice and the animals were monitored for diabetes for more than 36 weeks. Results showed that 7 of the 7 (100%) NOD recipient mice that had been injected with Stat5b-CA.BMDCs were protected against diabetes development. In marked contrast, 6 of the 7 (86%) of NOD recipient mice that had been injected with NOD.BMDCs developed diabetes over the period of observation (Figure 3). Together, these in vivo results, combined with those obtained using our in vitro BMDC system, led support to the critical role of active Stat5b in programming the tolerogenic function of BMDCs of NOD mice.

### 2.4. Stat5b-CA Differentially Regulates IRF4 and IRF8 Expression in BMDC of NOD Mice

**Figure 4:**
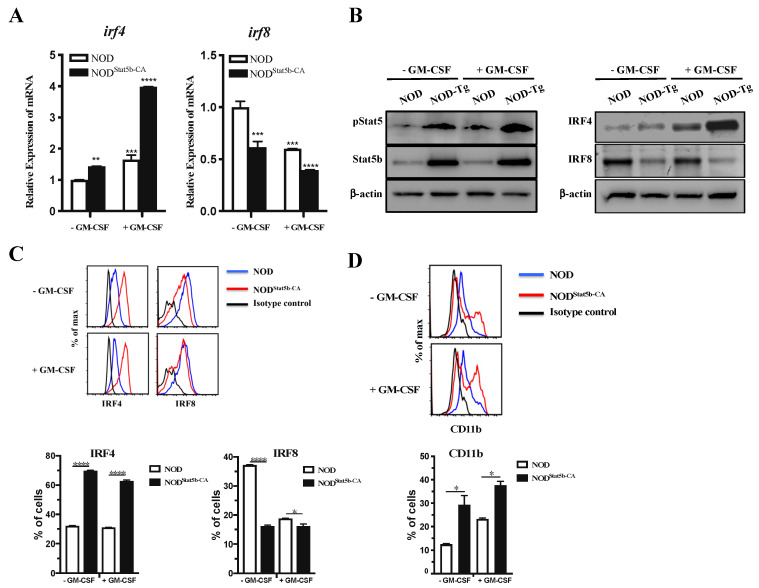
IRF4 and IRF8 expression is differentially regulated in Stat5b-CA-expressing BMDCs. BMDCs derived from NOD and NOD.CD11c^Stat5b-CA^ mice were cultured for 48 h in the absence or presence of GM-CSF (50 ng/mL). (**A**) *Irf4* and *Irf8* mRNA expression levels were determined by qRT-PCR using the ΔΔCT method. The data are shown as relative expression compared to untreated BMDCs derived from NOD mice. (**B**) Representative Western blot analysis of IRF4, IRF8, pStat5, and Stat5b expression. β-actin expression is shown as a gel-loading control. (**C**–**D**) Representative flow cytometry analysis of (**C**) IRF4 and IRF8, and (**D**) CD11b in CD11c^+^ BMDCs. Data are shown as the mean ± SEM of at least three independent experiments. The asterisks indicate statistically significant differences determined by one-way ANOVA with Tukey’s post-test. *p* < 0.05 (*), *p* < 0.01 (**), *p* < 0.001 (***), and *p* < 0.0001 (****).

The molecular details of the mechanism by which Stat5b-CA affects the BMDC transcriptional network driving their tolerogenic and immunogenic functions is not known. Recent reports have indicated that Stat5 influence DC subset development and function through regulation of IRF4 and IRF8 [12,13]. Moreover, IRF4 expression in DCs exerts its effects on T-cell differentiation toward Th2 responses, whereas IRF8-expressing DCs express more IFN-γ and IL-12 that promote Th1 response [14,15]. In this context, we have reported that CD4^+^ T cells educated with splenic DCs expressing Stat5b-CA exhibited a Th2-like immune response [9]. Therefore, we investigated whether IRF4 and IRF8 transcription factors were differentially regulated in Stat5b-CA.BMDCs and BMDCs of NOD mice. Real-time PCR analysis showed higher *irf4* and lower *irf8* gene expression in unstimulated Stat5b-CA.BMDCs than in NOD BMDCs (Figure 4A). *irf4* gene expression was highly upregulated, whereas *irf8* gene expression was downregulated in stimulated Stat5b-CA.BMDCs in comparison to NOD BMDCs (Figure 4A). As expected, unstimulated and GM-CSF-stimulated Stat5b-CA.BMDCs express high levels of pStat5 and Stat5b as compared to BMDCs of NOD mice (Figure 4B). Furthermore, Western blot (Figure 4B) and FACS analysis (Figure 4C) confirmed that IRF4 was highly expressed and that IRF8 expression was significantly reduced in Stat5b-CA.BMDCs compared to NOD BMDCs. Since it has been shown that IRF4 plays a critical role in the development of the CD11b^+^CD8α^−^ DC subset [16,17,18,19], we determined the level of expression of the CD11b^+^ marker in Stat5b-CA.BMDCs. FACS data showed that Stat5b-CA.BMDCs contained a higher percentage of CD11b^+^ DCs subset than NOD BMDCs (Figure 4D). To confirm these data, BMDCs from NOD and NOD.CD11c^Stat5b-CA^ mice were analyzed using Flt3L BM-derived DCs where representative DC subset (cDC1: CD11c^+^CD11b^−^; cDC2: CD11c^+^CD11b^+^; and pDCs: CD11c^+^B220^+^) differentiation occurs. Results (Supplementary Figure S1) showed a great reduction in DC1 subsets and increased DC2 subsets in Stat5b-CA.BMDCs as compared to NOD.BMDCs. The results showed also a great reduction of pDCs in Stat5b-CA.BMDCs, thereby corroborating the importance of Stat5 signaling in the inhibition of pDC development by suppressing the IRF8 transcription factor. Taken together, these data clearly indicated that the active form of Stat5b enhanced IRF4 and decreased IRF8 expression, leading to the enhanced development of a CD11c^+^CD11b^+^ subset in bone marrow-derived DCs.

### 2.5. The Active Form of Stat5b Recruits Ezh2 to Repress IRF8 but Not IRF4 Expression

**Figure 5:**
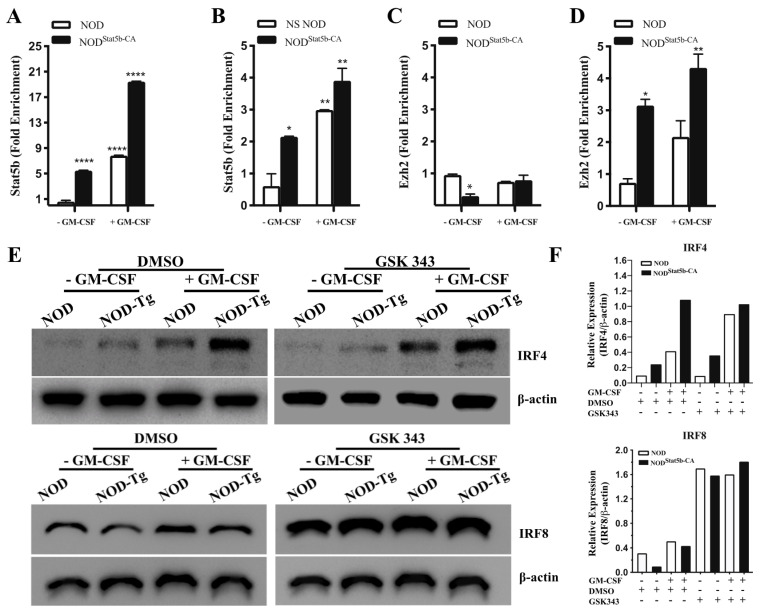
Ezh2 recruitment to upstream sequences of *Irf8* gene but not to the promoter of *Irf4* genes in BMDCs of NOD and NOD.CD11c^Stat5b-CA^ mice. BMDCs derived from NOD and NOD.CD11c^Stat5b-CA^ mice were cultured in the absence or presence of GM-CSF (50 ng/mL) for 48 h. ChIP experiments were performed using antibodies against (**A**,**B**) Stat5b or (**C**,**D**) Ezh2. DNA fragments of *Irf4* (**A**,**C**) and *Irf8* (**B**,**D**) were quantified by qPCR. IgG was used as a negative control. (**E**,**F**) BMDCs were pre-incubated for 1 h with the Ezh2 inhibitor GSK343 (3 µM) or vehicle (0.1% DMSO) prior to cultures being continued for 48 h in the absence or presence of GM-CSF (50 ng/mL). (**E**) Expression of IRF4 and IRF8 was analyzed by Western blot. (**F**) Quantitative data in Figure 5E normalized to loading control (β-actin). Data are shown as the mean ± SEM. The asterisks indicate statistically significant differences determined by one-way ANOVA with Tukey’s post-test. *p* < 0.05 (*), *p* < 0.01 (**), and *p* < 0.0001 (****).

The molecular mechanisms by which Stat5b-CA regulates expression of IRF4 and IRF8 in BMDCs and, consequently, their immunogenic as opposed to tolerogenic function remain to be established. The optimal binding motif for Stat5 has been defined as the GAS motif TTCN_3_GAA. While the dimeric form of Stat5 binds strongly to the GAS canonical motif, the tetrameric form of Stat5 binds to two GAS motifs [20]. Database analysis of IRF4- and IRF8-encoding genes has revealed that the *Irf8* gene contains two GAS motifs upstream of the 5′ end, whereas the *Irf4* gene contains only one GAS motif in that region. In addition, it has been reported that tetrameric Stat5 binding to the intronic *Igκ* enhancer Eκi, which recruits Ezh2, results in the repression of *Igκ* germline transcription during B cell lymphopoiesis [21]. To investigate whether Stat5b recruited Ezh2 to bind single or double GAS motifs upstream of the *Irf4* and the *Irf8* genes, respectively, we performed quantitative chromatin immunoprecipitation (ChIP) experiments using antibodies directed against Stat5b or Ezh2, followed by PCR amplification using primers specific for *Irf4* and *Irf8*. Results showed that Stat5b bound to the promoter of *Irf4* and to the enhancer of *Irf8* in Stat5b-CA.BMDCs and NOD BMDCs (Figure 5). However, DNA binding of Stat5b to the promoter of *Irf4* (Figure 5A) and *Irf8* (Figure 5B) was significantly increased in Stat5b-CA.BMDCs with respect to NOD BMDCs. The consequences of Stat5-mediated gene repression or activation vary depending on the context in which Stat5 binding occurs. We thus investigated whether Stat5-CA recruited Ezh2 to the GAS motifs of *Irf4* and *Irf8* upstream sequence. Results showed that the pull-down of Ezh2 resulted in the enrichment and the amplification of the *irf8* gene (Figure 5D), whereas no *irf4* gene amplification was detectable (Figure 5C). These results suggest that Ezh2 was not recruited to the *Irf4* promoter, whereas it was recruited to DNA fragments corresponding to the enhancer of *Irf8*. To confirm whether concomitant recruitment of Ezh2 and Stat5-CA to GAS sequences in the *Irf8* enhancer in Stat5-CA.BMDCs contributed to IRF8 repression, Stat5b-CA.BMDCs and NOD BMDCs were pre-incubated with the selective Ezh2 inhibitor GSK343. DCs were left unstimulated or were exposed to GM-CSF, and IRF8 and IRF4 expression was determined by Western blot. Results showed higher levels of IRF4 expression in Stat5b-CA.BMDCs than in NOD BMDCs, whereas IRF8 expression was lower in NOD BMDCs than in Stat5b-CA.DCs (Figure 5E,F). However, treatment with Ezh2 inhibitor GSK343 enhanced the expression of IRF8 in Stat5b-CA.DCs as well as in NOD BMDCs (Figure 5E,F). Interestingly, levels of IRF4 remained higher in Stat5b-CA.BMDCs as compared to NOD BMDCs (Figure 5E,F). These observations suggested that Ezh2 recruitment played a determining role in reducing IRF8 gene transcription but not in regulating IRF4 gene expression (Figure 5E). Altogether, these results suggested that active Stat5b recruited Ezh2 to bind to the IRF8 enhancer and repressed its transcription. Consequently, the absence of Ezh2 recruitment with Stat5b to the IRF4 promoter released IRF4 transcription in tolerogenic Stat5b-CA.BMDCs.

### 2.6. Ezh2 Inhibition in Tolerogenic Stat5b-CA.BMDCs Restores Their Immunogenic Function When Transferred to NOD Mice

**Figure 6:**
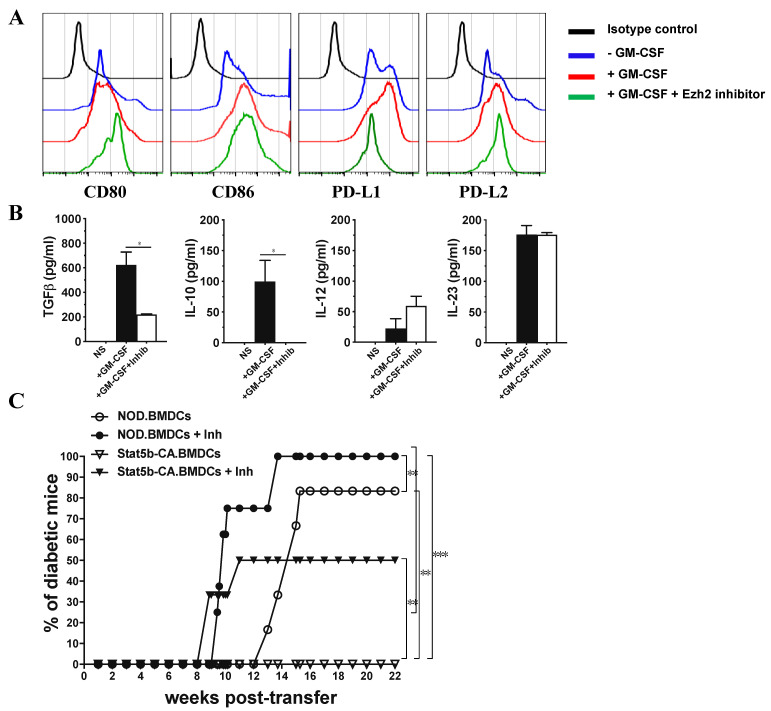
Inhibition of Ezh2 in Stat5b-CA-expressing BMDCs disturbs their tolerogenic signature and their capacity to protect against diabetes in NOD mice. (**A**,**B**) Stat5b-CA.BMDCs were pre-incubated for 1 h with the Ezh2 inhibitor GSK343 (3 µM) or vehicle (0.1% DMSO), then cultured for 48 h in the absence or presence of GM-CSF (50 ng/mL) and analyzed for (**A**) the expression of CD80, CD86, PD-L1, and PD-L2 by FACS and (**B**) production of cytokines (TGFβ, IL-10, IL-12, and IL-23) by ELISA. (**C**) BMDCs derived from NOD or NOD.CD11c^Stat5b-CA^ mice were cultured in vitro with Ezh2 inhibitor GSK343 (3 µM) or vehicle (0.1% DMSO). DCs were washed and i.v. injected (10^7^ cells/mouse) to 8-week-old female NOD mice (6 mice per group). Recipient NOD mice were followed for diabetes development until 26 weeks (22 weeks post-injection) of age. The asterisks indicate statistically significant differences determined by one-way ANOVA with Tukey’s post-test. *p* < 0.05 (*). For diabetes development, data were analyzed by Gehan–Breslow–Wilcoxon test. *p* < 0.01 (**) and *p* < 0.001 (***).

To examine whether Ezh2 is crucial for tolerogenic properties of Stat5b-CA.BMDCs, Stat5b-CA.BMDCs were treated in vitro with Ezh2 inhibitor GSK343 and analyzed for the expression of co-stimulatory and co-inhibitory markers and production of pro-inflammatory and anti-inflammatory cytokines. Results (Figure 6A,B) showed that Stat5b-CA.BMDCs that were pretreated with Ezh2 inhibitor prior to stimulation with GM-CSF upregulated the expression of CD80 and downregulated PD-L1 expression, whereas the expression of CD86 and PD-L2 remained unchanged. In addition, cytokine quantification showed that Ezh2 reduced TGFβ and IL-10, slightly enhanced IL-12, and had no effect on IL-23 secretion. These results suggested that the Ezh2 inhibitor switched Stat5b-CA.BMDC tolerogenic properties towards immunogenic properties.

To determine whether Ezh2 is crucial for tolerogenic function of Stat5b-CA.BMDCs, Stat5b-CA.BMDCs were treated in vitro with Ezh2 inhibitor GSK343 prior to their transfer to 8-week-old prediabetic NOD mice and following them for diabetes. Results (Figure 6) showed that NOD mice transferred with BMDCs of NOD mice became diabetic within 12 weeks after transfer, whereas the transfer of Stat5b-CA.BMDCs protected NOD mice from diabetes. Interestingly, injection of Ezh2 inhibitor GSK343-treated BMDCs into NOD mice accelerated diabetes onset in recipient NOD mice. Interestingly, the transfer of Stat5b-CA.BMDCs that had also been pretreated with Ezh2 GSK343 inhibitor to NOD mice restored and accelerated diabetes transfer in 50% of the recipient NOD mice. Together, these results suggest that Ezh2 participates in the tolerogenic function of DCs, thus limiting autoimmune development in NOD mice.

## 3. Discussion

Autoimmune diabetes in the NOD mouse model results, in part, from a dysfunction of DCs, which leads to a breakdown of self-tolerance [22,23,24], a key event in the mechanism’s underlying onset of autoimmune diseases. DCs can be reprogrammed to induce immune suppression under specific conditions. To overcome the Stat5 defect in DCs of diabetes-prone NOD mice, we generated transgenic mice (NOD.CD11c^Stat5b-CA^) expressing a DC-specific, constitutively active form of the *Stat5b* gene. This transgenic model allowed us to define the critical role of Stat5b as a transcription factor in reprogramming immunogenic to tolerogenic DCs [9]. In the present study, we investigated the molecular mechanisms by which active Stat5b induced tolerogenic DC function. We found that Stat5b-CA.BMDCs exhibited a mature phenotype, expressed high levels of PD-L1 and PD-L2, and produced large amounts of TGF-β but low amounts of TNFα and IL-23. Interestingly, in Stat5b-CA.BMDCs, Stat5b and the histone methyltransferase Ezh2 bind to the IRF8 enhancer to repress IRF8 transcription, whereas Stat5b but not Ezh2 binds to the IRF4 promoter to enhance IRF4 transcription. We also observed that Ezh2-dependent IRF8 repression and Ezh2-independent IRF4 upregulation led to increased CD11c^+^CD11b^+^ DC2 subset differentiation in Stat5b-CA.BMDCs. Of significance, injection of Stat5b-CA.BMDCs into diabetes-prone NOD mice halted ongoing diabetes, in marked contrast to reduced diabetes protection in NOD mice injected with Stat5b-CA.BMDCs in which Ez2 was inhibited.

The *Stat5b* gene is associated with the *idd4* susceptibility locus in NOD mice [25]. Furthermore, it has also been reported to be associated with several defects in NOD mice, such as weak DNA binding and reduced expression of downstream genes [8]. We have shown that some Stat5b defects could be overcome in transgenic NOD mice expressing a constitutively active form of diabetes resistant C57BL/6 Stat5b in DCs by promoting the tolerogenic function of mature splenic DCs, which we found to be critical in inducing and maintaining immune tolerance in autoimmune diabetes [9]. Similarly, bone marrow-derived Stat5b-CA.BMDCs also displayed a fully mature phenotype and expressed high levels of the PD-L1 and PD-L2 inhibitory molecules that are known to interact with PD-1 on T cells and to lead to inhibition of potentially diabetogenic peripheral T cells that have avoided negative thymic selection [26,27,28]. Furthermore, Stat5b-CA.BMDCs may also promote Treg development and their immunoregulatory function to prevent autoimmunity [29,30].

Several lines of evidence indicate that phenotypically mature DCs that display high expression levels of co-stimulatory molecules do not induce Th1 responses in every instance but promote instead Th2 immune response and/or Treg differentiation, depending on their cytokine profile [31]. Cytokine profiles of Stat5b-CA.BMDCs have revealed enhanced secretion of TGFβ and low production of TNFα and IL-23. It has been reported that TNFα and IL-23 produced by DCs are pathogenic in several autoimmune disorders [32,33]. IL-23 is one of the essential factors required for survival and/or expansion of Th17 cells, which produce IL-17, IL-17F, IL-6, and TNFα [33,34]. Tolerogenic DCs also play an important role in T-cell tolerance mediated by anti-inflammatory cytokine TGFβ [35], which is important for the maintenance and survival of Tregs [36], and inhibition of the induction and synthesis of pro-inflammatory cytokines such as IL-12p70 [37]. Indeed, we showed here that TGFβ-secreting Stat5b-CA.BMDCs produced lesser amounts of IL-12 than NOD BMDCs. Thus, unlike NOD BMDCs, Stat5b-CA-BMDCs were reprogrammed to secrete fewer pro-inflammatory cytokines and to increase production of TGFβ that represents the signature of tolerogenic DCs.

Recent studies have reported that Stat5 influences the development and function of DC subsets by controlling the regulation of IRF4 and IRF8 gene expression [12,13]. In mice, conventional DC subsets CD11b^−^ (DC1) or CD11b^+^ (DC2) arise also through distinct networks of transcription factors involving IRF4 and IRF8 and are specialized for unique functional responses [38]. It has been shown that IRF8 was necessary for the development of CD11c^+^CD11b^−^ resident and migratory DC subsets, whereas IRF4 was critical for the generation of CD11c^+^ CD11b^+^ DCs subsets [18,19,39]. In addition, the variation in IRF4 or IRF8 levels has an important role in regulating the magnitude of DC functional responses [40]. Consistent with these reports, data reported here showed that Stat5b-CA upregulated *Irf4* while downregulating *Irf8* gene expression in BMDCs. A large proportion of Stat5b-CA.BMDCs comprised a higher proportion of CD11c^+^CD11b^+^ DC2 as compared to the proportion of CD11c^+^CD11b^+^ DC2 subsets in NOD BMDCs. Our results also showed that tolerogenic Stat5b-CA.BMDC downregulated *Il12a*, *Il27a*, and *Il23a* gene expression that is involved in the induction of the Th1 [41,42,43] and Th17 immune responses [44,45,46,47], respectively. Our findings are consistent with previous reports that showed that IRF4 negatively regulates the production of pro-inflammatory cytokines in response to TLR ligands in macrophages [48,49] and that IRF8-expressing DCs are important for Th1 and CD8^+^ T cell responses [50,51]. However, IRF4-expressing DCs have also been found to be important for DC-driven polarization of mucosal and lung Th17 responses [16,17], for the induction of Th2 responses, and for the attenuation of Th1 responses [52].

Upstream sequences of the *Irf4* and *Irf8* genes contain a single GAS motif in the *Irf4* promoter region and two GAS motifs separated by nine nucleotides in the *Irf8* enhancer region. Our data showed increased recruitment of Stat5b to *Irf4* and *Irf8* GAS motifs and differential regulation of *Irf4* and *Irf8* gene expression in Stat5b-CA.BMDCs. These results can be explained either by the overexpression of Stat5b-CA and/or by the fact that Stat5b-CA is constitutively phosphorylated. In addition, previous studies have identified, in NOD mice, a mutation (L327M) at the DNA-binding domain of *Stat5b*, which alters Stat5b DNA-binding activity [8,53]. In agreement with these reports, our results showed a weak DNA-binding activity of Stat5b in NOD BMDCs. Furthermore, data reported here showed that the expression of Stat5b-CA of diabetes-resistant C57BL/6 mice restored strong Stat5b DNA-binding activity in the BMDCs of NOD mice. Investigating the mechanism by which Stat5b upregulated *Irf4* and downregulated *Irf8* gene expression revealed that Ezh2 was recruited by Stat5b and that the complex strongly bound to the DNA sequence region containing two GAS motifs in the *Irf8* enhancer. In contrast, Ezh2 was not recruited by Stat5b in the region that contains a single GAS motif at the *Irf4* promoter. Therefore, the recruitment of the Stat5b/Ezh2 complex may explain the low levels of expression of IRF8 in Stat5b-CA.BMDCs. In addition, our data are in agreement with the report that the tetrameric form of Stat5 interacts with Ezh2 at the locus κ (*igk*) to induce repression of transcription of the immunoglobulin genes following changes in chromatin and methylation of histone H3 at lysine 27 (H3K27) [21]. We further confirmed the involvement of Ezh2 in the recruitment by Stat5b as a mechanism of downregulation of *Irf8* gene expression by using an Ezh2 pharmacologic inhibitor (GSK343) that led to the upregulation of *Irf8* gene transcription as a result of the inhibition of Ezh2.

Active Stat5 binds to the TTCN_3_GAA γ-interferon activated sequence (GAS) single or tandem motif as dimers or tetramers [20,54,55]. Moreover, *Stat5b* paralog appears to play a major role in the immune system [55,56]. A recent report has shown that the dimerization of Stat5 was required for development of the immune system, whereas the tetramerization of Stat5 was critical for normal immune response. The authors also reported that Stat5 tetramers were required for Tregs to suppress experimentally induced colitis [55]. Therefore, uncovering the role of dimers or tetramers of Stat5b is crucial to understanding how they modulate signal transduction and how they perform their functions in DCs.

In conclusion, DCs represent a primary target for the development of therapeutics in many autoimmune diseases, including diabetes. In this context, our current findings clearly show that Stat5b is a key regulator of BMDC tolerogenic function. Our observations further highlight the fundamental role of Ezh2 in regulating IRF4 and IRF8 in tolerogenic DCs and limiting autoimmunity. Therefore, Ezh2 may represent a potential target to modulate the tolerogenic properties of DCs in the setting of autoimmune diseases.

## 4. Materials and Methods

### 4.1. Generation of Bone Marrow-Derived DCs

Bone marrow derived DCs were generated as we have reported [57,58]. Briefly, bone marrow cells were collected from femurs and tibias of transgenic NOD.CD11c^Stat5b-CA^ and NOD mice. One million cells were plated in 10 mL bacterial Petri dishes (UltiDent Scientific, St. Laurent, QC, Canada) and cultured in RPMI 1640 medium supplemented with 10% heat inactivated FBS, 100 U/mL penicillin, 100 μg/mL streptomycin, and β-mercaptoethanol (50 μM) in the presence of GM-CSF (5 ng/mL) and IL-4 (4.5 ng/mL) (R&D Systems Inc. Minneapolis, MN, USA). At day 3, fresh medium (10 mL) supplemented with GM-CSF (5 ng/mL) and IL-4 (4.5 ng/mL) was added to the culture. At day 5, half of the medium was removed and replaced with fresh medium supplemented with GM-CSF (5 ng/mL) and IL-4 (4.5 ng/mL). At day 7, non-adherent cells were gently harvested, pooled, and left unstimulated or exposed to GM-CSF (50 ng/mL) (R&D Systems Inc. Minneapolis, MN, USA) for 48 h. Over 92% of non-adherent cells were CD11c^+^. All experiments were performed with relevant guidelines and regulations. All mice were housed under pathogen-free conditions at the Faculty of Medicine in accordance with the guidelines of the Institutional Animal Care Committee of the University of Sherbrooke (Protocol # 93-18), Sherbrooke, QC, Canada.

### 4.2. Antibodies and Flow Cytometry Analysis

Analysis of unstimulated and stimulated BMDCs was done by staining the cells with the following antibodies: anti-CD11c-APC-Cy7 (clone N418: BioLegend, San Diego, CA, USA), anti-CD80-PE-Cy5 (clone 16-10A1), anti-CD86-PE-Cy5 (clone GL1), anti-CD40-PE-Cy5 (clone 1C10), biotin anti-MHC II streptavidin-PerCP (clone 39-10-8), anti-CD11b-PE (clone M1/70), anti-PD-L1-PE (clone MIH5), anti-B220 (clone RA3-6B2), or anti-PD-L2-PE (clone TY25) (were from eBiosciences, San Diego, CA, USA) and anti-Sirpα-PerCp-efuor710 (P84: Invitrogen, Burlington, ON, Canada). Flow cytometry data were collected on a CytoFLEX instrument (Beckman Coulter, Brea, CA, USA) and analyzed using FlowJo 10.2 software (Tree Star Inc., Ashland, OR, USA).

### 4.3. Real-Time PCR

Gene expression was measured by quantitative PCR as reported [58,59]. Briefly, RNA was extracted from BMDCs using the Trizol reagent (Invitrogen, Burlington, ON, Canada) and reverse transcribed using Superscript II (Invitrogen) and OligodT (Promega, Madison, WI, USA). Real-time PCR reactions were performed in a volume of 25 µL containing 10 ng of cDNA and 1 µM of each forward and reverse primers, using a Quantitect SYBR Green qPCR kit (Qiagen, Montreal, QC, Canada) in a Rotorgene 3000 instrument (Corbett Research, Sydney, Australia). Primer sequences used are listed in Table 1. Amplification plots were generated using Rotorgene Amplification software v6.0 (Corbett Research), and relative gene expression changes were calculated using the 2^−ΔΔCt^ method and normalized using β-actin expression.

### 4.4. Western Blots

BMDCs were washed in cold PBS and resuspended in lysis buffer (Tris 50 mM, NaCl 0.15 M, DTT 1 mM, Triton X-100 1% (*v*/*v*)) containing protease and phosphatase inhibitors. Cell lysates were fractionated on 10% SDS-PAGE gels, transferred to nitrocellulose membranes (Hybond-ECL, Amersham Biosciences, Baie d’Urfé, QC, Canada) and incubated overnight with anti-IRF4-biotin (clone M17), anti-IRF8-biotin (clone C19) (Santa Cruz Biotechnology, Santa Cruz, CA, USA), anti-pStat5 (clone C11C5) (Cell Signaling Technology, Beverly, MA, USA), or anti-Stat5b (clone EPR16671) (Abcam, Cambridge, MA, USA), followed by appropriate secondary antibodies. Bands were revealed by enhanced chemiluminescence (GE Health Care Canada Inc., Oakville, ON, Canada).

### 4.5. Cytokine Quantification

Supernatants from untreated or GM-CSF-treated BMDC cultures were collected after 48 h of incubation and stored at −20 °C until use. TGFβ, TNFα, IL-10, IL-12p170, and IL-23 were quantified using ELISA assays (ThermoFisher Scientific, Waltham, MA, USA), following the supplier’s instructions.

### 4.6. Chromatin Immunoprecipitation (ChIP) Assays

Chromatin immunoprecipitates were processed using an EZ-ChIP kit, according to the manufacturer’s protocol (Upstate Biotechnology, Lake Placid, NY, USA). Immunoprecipitations of sonicated chromatins were performed using monoclonal anti-Stat5b (Cell Signaling, Danvers, MA, USA) or polyclonal anti-Ezh2 (clone 4905S) (Cell Signaling, Danvers, MA, USA) Abs or with isotype-matched control IgG Abs coupled to microbeads. Samples were subjected to real-time PCR of the regions containing the proximal GAS site in the promoter of *Irf4* and the enhancer of *Irf8*. Primers used were 5′-CTTGGGATCTGCAGAGAAGCTGTG-3′ (forward) and 5′-TTGGCGCGCACCATCCT-3′ (reverse) for the *Irf4* promoter and 5′-CGCCCCCGGAGTAAAGAGAG-3′ (forward) and 5′-GCTAATTGAGGAGCGGGAGGGG-3′ (reverse) for the *Irf8* enhancer. Fold enrichments were calculated using the ChIP signal as the fold increase in signal relative to background signal [60].

### 4.7. Mice Treatment with BMDCs

BMDCs derived from NOD.CD11c^Stat5b-CA^ transgenic mice or from control NOD mice were left untreated or treated with Ezh2 inhibitor GSK343 (3 μM for 1 h), washed, and transfused intravenously (10^7^ cells/mouse) to 8- to 10-week-old female NOD mice. Mice were monitored for diabetes development by urine glucose test using Uristix strips (Bayer, Minneapolis, MN, USA) and confirmed by measurement of blood glucose levels with an Accu-Check Advantage monitoring system (Roche Diagnostics, Indianapolis, IN, USA). Diabetes was monitored up to 36 weeks in the case of mice injected with Stat5b-CA.BMDCs or until diabetes was detected in the case of mice injected with BMDCs of NOD mice. The animals were considered diabetic following two positive Uristix readings and when blood glucose concentration was higher than 15 mmol/L.

### 4.8. Statistics

Statistical analyses were performed using GraphPad Prism 7.0a software (GraphPad Software Inc., La Jolla, CA, USA). Diabetes incidence was compared using the Gehan–Breslow–Wilcoxon test. Student’s *t*-test or one-way ANOVA tests were used for statistical analysis for in vitro data analysis. Differences were considered significant for *p* < 0.05.

## Figures and Tables

**Table 1:** List of the primers used for qPCR experiments.

Gene	Forward Primer	Reverse Primer
*Il12a*	CTCCTAAACCACCTCAGTTTGGCCAGGGTC	TAGATGCTACAAGGCACAGGGTCATCATC
*Il12b*	CACTCATGGCCATGTGGGAGCTGGAGAAAG	TCCGGAGTAATTTGGTGCCTTCACACCTCAG
*Il23a*	GCCCCGTATCCAGTGTGA	GCTGCCACTGCTGACTAG
*Il27a*	CTGTTGCTGCTACCCTTGCTT	CACTCCTGGCAATCGAGATTC
*Tgfb1*	TGACGTCACTGGAGTTGTACGG	GGTTCATGTCATGGATGGTGC
*Irf4*	TCGGCCCAACAAGCTAGAAA	GGCCATGGTGAGCAAACACT
*Irf8*	CGTGGAAGACGAGGTTACGCTG	GCTGAATGGTGTGTGTCATAGGC
*β-actin*	ACCCACACTGTGCCCATCTA	TCATGGATGCCACAGGATTC

## Supplementary Materials

The following are available online at https://www.mdpi.com/1422-0067/21/18/6453/s1.

## Abbreviations

DCs	dendritic cells
Stat5b-CA	constitutively active Stat5b
