# Supplementary material for: Elucidating the Role of Ezh2 in Tolerogenic Function of NOD Bone Marrow-Derived Dendritic Cells Expressing Constitutively Active Stat5b
Source: Int J Mol Sci. 2020 Sep 4;21(18):6453. doi: 10.3390/ijms21186453 (PMC7554732; doi:10.3390/ijms21186453)
Supplement: Supplementary file 1 [file ijms-21-06453-s001.pdf]

Supplementary figure S1

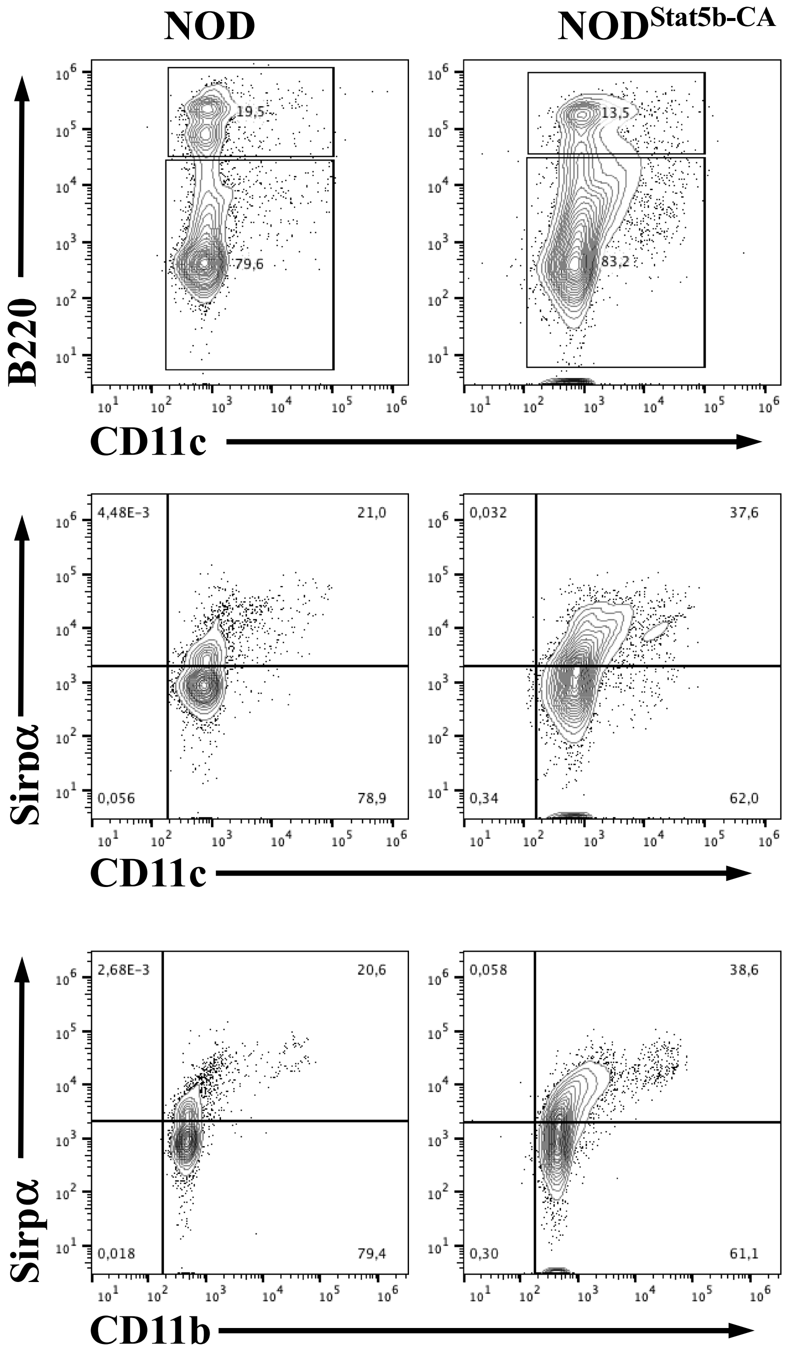

Figure S1. Percentages of Sirpα⁺-expressing BMDCs generated from NOD and NOD$^{Stat5b-CA}$ mice. BMDCs were derived from NOD and NOD.CD11c$^{Stat5b-CA}$ mice using Flt3L (50 ng/mL) for 6 days and analyzed by flow cytometry. Data are a representative FACS profile of CD11c+B220+ cells, CD11c+B220-Sirpα+ cells, and CD11c+ B220-CD11b+Sirpα+ cell subsets.
